# Prevalence of Cigarettes and Waterpipe Smoking among Jordanians, Refugees, and Migrants in Jordan and Its Associated Factors: A Secondary Data Analysis

**DOI:** 10.3390/ijerph20010082

**Published:** 2022-12-21

**Authors:** Osama Alkouri, Yousef Khader, Ahmad M. Al-Bashaireh

**Affiliations:** 1 Faculty of Nursing, Yarmouk University, Irbid 2116, Jordan; 2 Department of Public Health, Community Medicine, Jordan University of Science and Technology, Irbid 21163, Jordan; 3 Faculty of Health Science, Higher Colleges of Technology, Fujairah 23036, United Arab Emirates

**Keywords:** smoking, refugees, migrants, cigarettes, waterpipe, prevalence

## Abstract

Smoking is a major public health threat resulting in increased morbidity and mortality. This study aimed to determine the prevalence of current smoking among different adult populations in Jordan, including Jordanians, refugees, and migrants and determine the factors associated with smoking. A cross-sectional survey was conducted during the period of June–September 2021 among Jordanians, Syrian refugees (both urban and camp refugees), and migrants living in four governorates in Jordan, including Amman, Zarqa, Mafraq, and Irbid. The selection of the four governorates was based on criteria related to the presence of Syrian refugees in host communities. The highest prevalence of cigarette smoking was among urban Jordanians (51.1%), followed by non-camp refugees (46.7%), while the highest prevalence for waterpipe (43.2%) and dual smoking (28.7%) was among non-camp refugees. Being male, aged 25–45 years old, unemployed, and having less than a high school education, as well as being urban Jordanians, were all associated with higher odds of cigarette smoking. Being male, unemployed, and urban Jordanians or non-camp refugees were variables associated with higher odds of waterpipe smoking. The study findings indicate that the identification of smoking prevalence in Jordan and its associated factors, including gender, educational level, employment status, regional area, migration, and exposure to traumas, should be considered by healthcare providers to formulate smoking cessation programs tailored to their needs and ultimately reduce smoking prevalence.

## 1. Introduction

Smoking is a major public health threat resulting in increased morbidity and mortality [1]. Worldwide, nearly 1.3 billion people aged 15 years and above are current smokers, 80% of whom live in low- and middle-income countries where the illness and death-related burden of tobacco is heaviest [2]. Plantations and the consumption of tobacco have increasingly grown in developing countries [1]. The number of smokers in developing countries is expected to rise due to various reasons, including population growth, increased affordability of tobacco, income growth, and aggressive marketing in those countries [1]. A study report showed that smoking among adolescents in low-middle income countries (LMICs) makes up more than four times the level in the United Kingdom [1].

In Jordan, the epidemic of smoking is rising significantly and is considered a public health emergency [3]. The estimated prevalence of smoking among adult males is 70.2% [4], which is the topmost in the Eastern Mediterranean Region and the second topmost in the world, after Indonesia. Additionally, smoking is markedly prevalent among Jordanian youths aged 13–15 years, 45% of which have made an attempt to smoke a tobacco product [5,6]. Smoking also has a significant impact on second-hand smokers, involving 68% of adults and 62% of youth who are exposed to second-hand smoke [7].

Smoking can take several forms, including cigarettes, cigars, waterpipes, tobacco products, cigarillos, pipe tobacco, and electric cigars. All of these forms are considered deleterious and unsafe [8]. In Jordan, people consume tobacco in two main ways: cigarettes and waterpipes [7]. The number of individuals smoking waterpipes is rising dramatically, particularly among the youth [9,10,11,12].

Various factors have been found to affect smoking status, including age, socioeconomic status, level of education, peer smoking, the attitudes of family and friends, lifestyle, stress, self-esteem, and health concerns [13,14,15,16,17]. A survey study among 3658 adults in five countries, including China, Jordan, India, Taiwan, and Saudi Arabia, showed that males, adults older than 25 years, and those working in the medical field were more likely to smoke [16]. Another study from Saudi Arabia showed that being married, in financial hardship, believing that a waterpipe is less harmful than a cigarette, and having a waterpipe-smoking sibling were the predictors of smoking waterpipes, cigarettes, and tobacco in the past 30 days [18].

Importantly, migration and exposure to conflict/traumas, variables experienced by refugees, were shown to affect smoking status [19,20]. Jordan has a large number of migrants and refugees. Nearly 670,637 Syrian refugees arrived in Jordan due to crises, leading to a regional humanitarian emergency, and most of them are living in urban areas [21]. In addition to the Syrian refugee influx, a large number of people migrated to Jordan in 2019 to escape areas of conflict, including 6000 Sudanese, 15,000 Yemenis, 67,000 Iraqis, and 2500 refugees from other nationalities [22]. While different studies reported prevalence rates of smoking among Jordanians, there remains a scarcity of studies assessing the prevalence of smoking among vulnerable populations in Jordan, including refugees and migrants. The evidence regarding smoking status and its associated factors among vulnerable groups is still contradictory and limited [23]. In addition, the generalizability of findings from studies in Jordan is limited, and the samples did not represent subpopulations in Jordan. Ignoring the problem of smoking among refugees and migrants tends to compromise the tobacco control efforts in Jordan. Therefore, this study aimed to determine the prevalence of current smoking among different adult populations in Jordan, including Jordanians, refugees, and migrants, and the factors associated with smoking.

## 2. Materials and Methods

### 2.1. Study Design

This study is based on a secondary analysis of recent data collected primarily to assess tuberculosis-related knowledge, behaviors, attitudes among refugees, migrants, and the general population in Jordan. The primary study was a cross-sectional study of rural Jordanians, urban Jordanians, non-camp refugees, camp refugees, and migrants living in the four major governorates of Amman, Zarqa, Mafraq, and Irbid. The study was conducted during the period of June–September 2021. The selection of these four governorates was based on criteria related to the presence of Syrian refugees in host communities. Almost 89.7% of Syrian refugees live in these governorates; 198,148 (29.6%) in the Amman governorate, 167,191 (25%) in Mafraq, 136,820 (20.4%) in Irbid, and 98,254 (14.7%) in Zarqa [13]. In total, 131,300 Syrian refugees live in camps. The number of Jordanians in these governorates was 4,226,700 (42.0% of Jordan’s population) in Amman, 580,000 (5.8%) in Mafraq, 1,867,000 (18.6%) in Irbid, and 1,439,500 (14.3%) in Zarqa.

### 2.2. Sampling

For Jordanians, a two-stage cluster sample of households was selected using the 2015 census frame. Each of the governorates of Jordan is divided into progressively smaller districts, sub-districts, localities, areas, and sub-areas. In addition to these administrative units, in the recent population census, each sub-area was divided into convenient area units called census blocks. An electronic file contains a complete list of all census blocks, with information on households, populations, and the geographical location of each block. The census blocks are regrouped to form a general statistical unit of moderate size, called a cluster, which is widely used in various surveys as the primary sampling unit. The sampling units in the first stage included clusters in the four governorates. The second stage of household selection involved choosing a random sample of households from each cluster using a systematic sampling technique. For each selected household, one eligible adult resident (≥18 year old) was randomly selected.

For urban Syrian refugees, the UNHCER databases were used to identify clusters of Syrian refugees in each governorate. A random sample of clusters was selected. From each selected cluster, a systematic random sample of households was selected. For Syrian camp refugees, a systematic random sample of households/prefabs was selected from each district in the Zaatari camp.

Migrants were identified as those attending the health centers for obligatory testing for the purpose of work permits. Additionally, a sample of migrants was selected after mapping the places where migrants work (agricultural, industrial, factories, etc.) in the four governorates. A cluster sample of work settings and migrants within these settings was randomly selected.

### 2.3. Data Collection

For data collection, a structured questionnaire was used to collect the data face-to-face. For host communities, urban refugees, and migrants, trained research assistants visited the selected households in the four governorates. For each selected household, one eligible adult resident (≥18 year old) was randomly selected to fill the survey. A household was visited at least twice before we marked it as ‘not at home’. If the house was uninhabited, the next household was selected. For camp refugees from the Zaatari camp, three trained research assistants, who were Syrian activists with a medical background, visited the selected households/prefabs and interviewed one eligible adult from each household.

### 2.4. Study Questionnaire

A structured questionnaire was used to collect data. The first part of the questionnaire comprised nine items of socio-demographic data, including age, gender, marital status, level of education, household classification, nationality, residential governorate, employment, and if they borrowed money over the last month to cover expenses and/or their bills. The second part of the questionnaire comprised two items about current cigarette and waterpipe smoking. Other sections of the questionnaire collected information on knowledge, attitude, and practices related to tuberculosis. Only the variables in the first and second sections were analyzed for the purpose of this study.

### 2.5. Ethical Considerations

This study followed the guidelines of the Declaration of Helsinki. This study was approved by the Institutional Review Board (IRB) of the Ministry of Health (MOH). Before the enrollment of participants, researchers and research assistants asked all participants to sign a consent form that clearly stated the purpose of the study, potential risks and benefits, the voluntariness of participation, and the right to discontinue participation at any time without any repercussions. All of the data were kept in a locked area and were only accessed by the principal investigator. Official permission was obtained from the principal investigator to use the data for estimating the prevalence of smoking.

### 2.6. Statistical Analysis

The data were analyzed using IBM SPSS version 22 (IBM: Armonk, NY, USA). All of the categorical data were reported as frequencies and percentages, and all numerical data were reported as averages and standard deviations. The chi-square test was used to compare the prevalence rates of smoking. Logistic regression was used to examine the associations between cigarette and waterpipe smoking and the participants’ demographics and their relevant characteristics. The interaction terms between groups and other studied characteristics were tested, and none were significant. A *p*-value equal to or less than 0.05 was considered statistically significant.

## 3. Results

### 3.1. Participants’ Characteristics

A total of 4574 persons (232 rural Jordanians, 1077 urban Jordanians, 1183 non-camp refugees, 1008 camp refugees, and 1074 migrants) were included in the study. Table 1 shows the participants’ demographics and relevant characteristics. Almost 53.6% of the sample were males, 58.6% were aged between 24 and 45 years, 68.8% were married, and 40% had not completed their high school education.

### 3.2. Prevalence of Smoking

Table 2 shows the gender-specific prevalence rates of current cigarette smoking, waterpipe smoking, and dual smoking among rural Jordanians, urban Jordanians, non-camp refugees, camp refugees, and migrants. Overall, the highest prevalence of cigarette smoking was among urban Jordanians, followed by non-camp refugees (51.1%, and 46.7%, respectively). The highest prevalence for waterpipe and dual smoking was among non-camp refugees, followed by urban Jordanians (waterpipe use: 43.2% and 41.3%, respectively; dual-use: 28.7% and 26.5%, respectively). Males in all groups had a higher prevalence of cigarette and dual smoking than females. The prevalence of waterpipe use was higher among males for rural Jordanians, non-camp refugees, and camp refugees and higher among females for urban Jordanians and migrants.

### 3.3. Factors Associated with Cigarettes and Waterpipe Smoking

Table 3 shows the multivariate analysis of factors associated with cigarette and waterpipe smoking. Jordanians living in urban areas were 1.65 times more likely than Jordanians living in rural areas to be cigarette smokers (OR: 1.7, 95% CI: 1.2–2.3, *p* = 0.005). Jordanians and refugees living in urban areas were 1.46 times and 1.54 times more likely than Jordanians living in rural areas to be waterpipe smokers (OR: 1.5, 95% CI: 1.1–2.0, *p* = 0.024; and OR: 1.5, 95% CI: 1.1–2.1, *p* = 0.009, respectively). Males were more likely than females to be cigarette smokers (OR: 4.3, 95% CI: 3.7–4.9, *p* = 0.003) and waterpipe smokers (OR: 1.5, 95% CI: 1.3–1.7, *p* < 0.001). Compared to participants aged younger than 25 years, those who were aged between 25 and 45 years old were more likely to be cigarette smokers (OR: 1.3, 95% CI: 1.2–1.7, *p* < 0.001), and those older than 45 years were more likely to be waterpipe smokers. The odds of cigarette smoking and waterpipe smoking varied significantly according to the governorate. Those who were unemployed were more likely to smoke cigarettes and use waterpipes.

## 4. Discussion

This study showed that the highest prevalence rates of cigarette and waterpipe smoking were among urban Jordanians. This finding can be explained by the unplanned settlement in urban areas that may increase social isolation, changes in lifestyle, and inequality among residents. This high prevalence ultimately results in an increased non-communicable disease burden [24]. Therefore, social and physical changes experienced in the environment of urban areas may result in promoting an unhealthy lifestyle and behaviors such as smoking [24]. This finding is in line with other studies [20,25,26]. Additionally, Jordanian people living in the cities with a high socioeconomic level may then be more likely to be exposed to second-hand smoke, their smoking habits are easily affected by their friends smoking, and they are more likely to have the financial capability to afford smoking costs [27,28,29].

Living in rural areas can also be associated with unemployment, psychological distress, lower levels of education, and decreased accessibility to healthcare, all of which ultimately increase smoking rates [30]. Furthermore, the inadequate implementation of smoking control-related policies can explain the high prevalence of smoking among refugees [30].

Lastly, the majority of Syrian refugees in Jordan are living in urban areas (80.6%) [22]. This can be explained by the fact that exposure to traumas/ man-made disasters such as political unrest, armed conflict, and interpersonal victimization (such as that experienced by refugees) can heighten vulnerability and raise rates of smoking [19].

The current study showed that Jordanian males have a higher prevalence of smoking than Jordanian females. This finding is in line with a cross-sectional study that investigated smoking prevalence among 3196 Jordanian adults in relation to demographics, with an emphasis on four smoking products (cigars, cigarettes, waterpipes, and pipes). The smoking rate was significantly higher among males (54.9%) compared to females (8.3%). The most frequent type of smoking product among males was cigarette smoking, followed by waterpipe smoking [31]. A recent systematic review also reported higher smoking prevalence among males in several Arab countries compared to females Jordan (54.3% vs. 11.1%, *p* < 0.005), Syria, Palestine, Yemen, Egypt, Bahrain, Tunisia, and King of Saudi Arabia [32]. The higher prevalence of smoking among males can be attributed to the fact that smoking is not socially acceptable among females [32,33].

The current study found that people aged between 35 and 45 years are more likely to be cigarette smokers. This finding is incongruent with the results of previous studies conducted in both developing and developed countries [31,32,34,35]. The prevalence of smoking in middle adulthood can be attributed to socioeconomic status [31]. For example, in a cross-sectional study of 3196 Jordanian adults aged 18+ years to estimate smoking prevalence, the highest prevalence of smoking was among people aged between 40 and 49 years (40.0%) [35]. Furthermore, people with low education attainment (70.5%), low income (below USD 430) (50%), and those who experienced social/family detachment (such as divorce, separation, widowhood) represented the majority of participants in this study [31]. The other explanation may include that smoking control policies were only focused on/tailored to adolescents, while people in middle adulthood were neglected [35]. Therefore, smoking surveillance and prevention should be expanded to include young adults.

The prevalence of waterpipe smoking across all nationalities of migrants was higher among females than males. Such findings were also extended to urban Jordanians. This might be attributed to the influence of marketing power, social media, encouraging environment in cafes, social activity during large gatherings, the freedom of females to access waterpipe tobacco as easily as males, and the belief that waterpipe smoking is less harmful than cigarette smoking [36,37]. Furthermore, the use of waterpipe tobacco for women is considered more socially acceptable compared to cigarette smoking, which ultimately increases the prevalence of waterpipe smoking among females in Arab countries [32]. The social acceptability of waterpipe tobacco may be associated with its sweet flavors and the presence of family members smoking waterpipes. Previous studies indicated that women prefer sweet-flavored tobacco due to its appealing smell and taste, and that increases their motivation to begin and continue waterpipe smoking. Despite the scarcity of information regarding possible health-related risks associated with flavored tobacco, new regulations to reduce flavored waterpipe tobacco needs to be implemented [32,33].

The present study found that the smoking of cigarettes and waterpipes is more prevalent among unemployed people compared to employed people. Unemployment can lead to increased levels of anxiety, stress, and psychological distress, which can be attributed to financial hardship and having the same daily routine [38]. The literature indicated that smoking has short-term benefits in terms of relieving stress and anxiety. Thus unemployed people may resort to smoking as a coping strategy to relieve stress and anxiety [38]. Previous studies showed an association between unemployment and a higher prevalence of smoking [39,40,41]. For example, in California, unemployed people who seek jobs were shown to have the highest prevalence of smoking (21%) compared to the employed (15%) [39]. Another example in the United Kingdom showed that smoking prevalence was two-fold higher among unemployed people compared to employed or those with financial hardship [40].

Our study found that participants with lower education had higher smoking prevalence compared to people with higher education. A study was performed to identify the association between cigarette smoking and education in four Arab countries (Jordan 2012 DHS, Palestine 2010 Family Health Survey, Lebanon 2004 PAPFAM, and Syria 2009 PAPFAM). The results showed that the prevalence of cigarette smoking among both genders from the four Arab countries is higher among people with limited access to education, which was determined to be a primary cause [42]. The center for Disease Control and Prevention (CDC) reported that the duration of smoking cigarettes among people with a high school education is two-fold longer than those with at least a bachelor’s degree [43]. The diffusion of innovation theory and fundamental cause theory can explain the higher prevalence of smoking among people with lower education [42]. The diffusion of innovation theory states that socially advantaged people can adopt new health practices and ideas, while the disadvantaged may take up these health practices later in the diffusion process [42].

The findings of the current study have several implications. Smoking in public places can be banned to minimize active smoking and exposure to second-hand smoke. The number of green public areas should also be increased, particularly in urban areas, which may allow individuals to perform physical exercises to relieve their stress rather than resorting to smoking. Smoking in Jordan can be reduced through the delivery of educational programs, implementing smoking control-related policies, improving people’s satisfaction, and reducing their stress, particularly in urban areas. Schools and universities should participate in educational programs by launching anti-smoking campaigns. Additionally, educational programs should aim to increase awareness about waterpipe smoking-related adverse effects, especially among females and young adults, to be presented through television and social media.

The findings of the present study should also draw the attention of public health professionals to the importance of tailoring community-based smoking cessation programs to smokers’ needs and socio-demographic characteristics (such as socioeconomic status, languages, ethnicity, level of education, gender, and cultural background). Healthcare providers need to be trained to deliver counseling regarding smoking cessation and identify smoking patterns among vulnerable groups (such as migrants and refugees) to raise awareness and perform culturally appropriate as well as gender-specific interventions. Lastly, further research studies are essential to identify the effectiveness of smoking prevention and cessation programs and to study the epidemiology of all smoking forms.

The strengths of this study include the analysis of data from large and nationally representative surveys to address the limited data regarding the prevalence rates of smoking cigarettes and waterpipes, especially among migrants and refugees. The study made comparisons in smoking prevalence rates across the study groups, including rural Jordanians, urban Jordanians, non-camp refugees, camp refugees, and migrants in Jordan. Our study has several limitations. Although our sampling approach for selecting Jordanians is similar and consistent with the 2017–2018 Jordan Population and Family Health Survey (JPFHS), only four governorates out of 12 were selected, and this, in fact, may affect the representativeness for the whole Jordanian population. The application of logistic regression is considered another limitation due to the stratification of the sample with undocumented and unequal sampling proportions.

## 5. Conclusions

The prevalence of cigarette and waterpipe smoking is high among Jordanians, refugees, and migrants, with rates being higher among urban Jordanians and non-camp refugees compared to rural Jordanians, camp refugees, and migrants. Unemployment and low education attainment were also associated with higher smoking prevalence. The study findings indicate that the identification of smoking prevalence in Jordan and its associated factors, including gender, educational level, employment status, regional area, migration, and exposure to traumas, should be considered by healthcare providers to formulate smoking cessation programs tailored to people’s needs. This will ultimately reduce smoking prevalence.

## Figures and Tables

**Table 1:** The socio-demographic characteristics of Jordanians, refugees, and migrants.

	Study Group					
	Jordanian Rural n (%)	Jordanian Urban n (%)	Non-Camp Refugees n (%)	Camp Refugees n (%)	Migrant n (%)	Total n (%)
Gender						
Male	115 (49.6)	552 (51.3)	551 (46.6)	463 (45.9)	769 (71.6)	2450 (53.6)
Female	117 (50.4)	525 (48.7)	632 (53.4)	545 (54.1)	305 (28.4)	2124 (46.4)
Age (year)						
<25	69 (29.7)	307 (28.5)	347 (29.3)	295 (29.3)	184 (17.1)	1202 (26.3)
25–45	118 (50.9)	627 (58.2)	656 (55.5)	558 (55.4)	723 (67.3)	2682 (58.6)
>45	45 (19.4)	143 (13.3)	180 (15.2)	155 (15.4)	167 (15.5)	690 (15.1)
Governorate						
Amman	42 (18.1)	519 (48.2)	434 (36.7)	4 (0.4)	371 (34.5)	1370 (30.0)
Mafraq	8 (3.4)	202 (18.8)	206 (17.4)	998 (99.0)	121 (11.3)	1535 (33.6)
Zarqa	12 (5.2)	226 (21.0)	188 (15.9)	2 (0.2)	373 (34.70	801 (17.5)
Irbid	170 (73.3)	130 (12.1)	355 (30.0)	4 (0.4)	209 (19.5)	868 (19.0)
Marital Status						
Single	107 (46.1)	417 (38.7)	403 (34.1)	267 (26.5)	234 (21.8)	1428 (31.2)
Married	125 (53.9)	660 (61.3)	780 (65.9)	741 (73.5)	840 (78.2)	3146 (68.8)
Education						
<high school	22 (9.5)	236 (21.9)	602 (50.9)	536 (53.2)	433 (40.3)	1829 (40.0)
Completed high school	66 (28.4)	314 (29.2)	309 (26.1)	283 (28.1)	298 (27.7)	1270 (27.8)
>high school	144 (62.1)	527 (48.9)	272 (23.0)	189 (18.8)	343 (31.9)	1475 (32.2)
Employment						
Employed	85 (36.6)	409 (38.0)	361 (30.5)	244 (24.2)	780 (72.6)	1879 (41.1)
Unemployed	62 (26.7)	341 (31.7)	471 (39.8)	403 (40.0)	186 (17.3)	1463 (32.0)
Student	85 (36.6)	327 (30.40	351 (29.7)	361 (35.8)	108 (10.1)	1232 (26.9)
Borrowed money to pay bills last month						
Yes	81 (36.5)	363 (35.5)	550 (48.8)	471 (48.3)	258 (24.3)	1723 (39.1)
No	126 (56.8)	539 (52.7)	428 (38.0)	429 (44.0)	699 (65.9)	2221 (50.4)
Don’t know	15 (6.8)	120 (11.7)	149 (13.2)	75 (7.7)	103 (9.7)	462 (10.5)

**Table 2:** The gender-specific prevalence rates of current cigarettes smoking, waterpipe smoking, and dual smoking among study groups.

	Group										*p*-Value (Group Difference)
	Rural Jordanians		Urban Jordanians		Non-Camp Refugees		Camp Refugees		Migrant		
	n	%	n	%	n	%	n	%	n	%	
Cigarettes											
Male	70	60.9	367	66.5	355	64.4	284	61.3	356	46.3	<0.001
Female	14	12.0	183	34.9	198	31.3	90	16.5	85	27.9	<0.001
Total	84	36.2	550	51.1	553	46.7	374	37.1	441	41.1	<0.001
*p*-value (gender difference)	<0.001		<0.001		<0.001		<0.001		<0.001		
Waterpipe											
Male	46	40.4	223	40.5	265	48.1	190	41.0	221	28.7	<0.001
Female	32	27.4	221	42.1	246	38.9	84	15.4	90	29.5	<0.001
Total	78	33.8	444	41.3	511	43.2	274	27.2	311	29.0	<0.001
*p*-value (gender difference)	0.037		0.589		<0.001		<0.001		0.802		
Dual smoking											
Male	34	29.6	175	31.7	208	37.7	160	34.6	175	22.8	<0.001
Female	11	9.4	110	21.0	132	20.9	57	10.5	48	15.7	<0.001
Total	45	19.4	285	26.5	340	28.7	217	21.5	223	20.8	<0.001
*p*-value (gender difference)	<0.001		<0.001		<0.001		<0.001		0.011		

**Table 3:** Factors associated with cigarettes and waterpipe smoking among participants.

	Cigarettes Smoking				Waterpipe Smoking			
Variable	OR	95% Confidence Interval		*p*-Value	OR	95% Confidence Interval		*p*-Value
Nationality								
Rural Jordanians	1				1.0			
Urban Jordanians	1.7	1.2	2.3	0.005	1.5	1.1	2.0	0.024
Non-camp refugees	1.4	1.0	1.9	0.074	1.5	1.1	2.1	0.009
Camp refugees	0.7	0.5	1.1	0.147	0.9	0.6	1.3	0.415
Migrants	0.9	0.6	1.2	0.394	0.9	0.6	1.3	0.546
Gender (male vs. female)	4.3	3.7	4.9	<0.001	1.5	1.3	1.7	<0.001
Age (year)								
<25	1.0				1.0			
25–45	1.4	1.2	1.7	<0.001	0.9	0.7	1.0	0.144
>45	1.2	0.9	1.5	0.132	0.8	0.6	1.0	0.040
Governorate								
Irbid	1.0				1.0			
Amman	1.9	1.5	2.3	<0.001	1.2	1.0	1.5	0.062
Mafraq	1.5	1.2	2.0	0.001	0.9	0.7	1.2	0.569
Zarqa	0.7	0.6	0.9	0.003	0.7	0.5	0.9	0.001
Marital status (single vs. married)	1.1	0.9	1.3	0.519	1.1	1.0	1.3	0.117
Education								
>high school	1.0				1.0			
<high school	1.2	1.0	1.4	0.036	0.9	0.7	1.0	0.076
High school	1.0	0.8	1.2	0.711	0.8	0.7	1.0	0.038
Employment								
Student	1.0				1.0			
Employed	1.1	0.9	1.4	0.176	1.1	0.9	1.3	0.273
Unemployed	1.2	1.0	1.4	0.05	1.2	1.0	1.4	0.023

## Data Availability

Data are available upon reasonable request.
